# Reviewed article: Bezerra EFS, Haddad MCF L, Moreira MAR, Gavioli A, Nogueira LS, Martins EAP. Analysis of mortality of users treated at a Mobile Emergency Care Service: an observational study, Paraná, 2019-2020. Epidemol Serv Saude. 2025:34;e20240092

**DOI:** 10.1590/S2237-96222025v34e20240092.a

**Published:** 2025-09-15

**Authors:** Betine Pinto Moehlecke Iser

**Affiliations:** 1 Universidade do Sul de Santa Catarina, Tubarão, SC, Brazil Universidade do Sul de Santa Catarina Tubarão SC Brazil

## Editor’s review

## First round comments

Caros autores 

Considerando as críticas e importantes apontamentos sobre o manuscrito apresentados, recomenda-se uma revisão majoritária no manuscrito e sua reapresentação, para reconsiderarmos a possibilidade de publicação na RESS. Sugere-se que revisem atentamente a todas as sugestões do corpo editorial e respondam item a item as revisões realizadas.

No texto em geral, especialmente nos resultados, precisaria rever a pontuação e espaçamento. O uso de siglas não consagradas dificulta o entendimento e por isso não é recomendado.

A discussão dos resultados esteve muito focada na ocorrência da pandemia de COVID-19. No entanto, o desfecho de interesse do estudo foi o óbito relacionado ao tempo transcorrido do chamado ao atendimento, e não foi realizada uma análise específica se o tempo diferiu em virtude da pandemia, o que as tabelas apresentam é uma diferença em relação ao óbito.

Outros detalhamentos, vide abaixo:

No resumo, salienta-se que a descrição do HR deve vir acompanhada do IC95%. Ressalta-se diferença para o ano 2020, ano da pandemia de COVID-19, no entanto não esta indicado qual o período de análise do estudo.

Quanto aos descritores, ´tempo de reação´ não parece adequado.

Se for manter o quadro de contribuições, destacar os principais resultados de forma sumária e com dados. O texto inserido não esta claro.

Métodos

Importante detalhar nos métodos como é feito o registro do tempo entre o chamado e o atendimento e suas possíveis falhas, pois esta é uma variável essencial para o estudo, suas aplicações e limitações.

Pagina 7 de 23: Linhas 25-28 o trecho “Além de mais um município que possui base descentralizada composta por apenas uma USB” parece deslocado. Linhas 41-43: “idade categorizada conforme preconizado pela Organização Mundial da Saúde (OMS)” indicar as categorias Linhas 14-18: Cita-se analise univariada de Regressao de Cox e, posteriormente, análise da “ razão de óbitos (por mil atendimentos) em associação com o tempo e Polo de atendimento foi utilizado o teste Chi-quadrado de Pearson”, de onde se entende que o tempo foi categorizado, sendo necessário indicar as categorias de tempo analisadas. Importante fornecer mais detalhes sobre a análise de sobrevida, indicando se há censuras.

Resultados

Os resultados são apresentados de maneira confusa, incluindo a formatação das tabelas. Aparecem dados referentes aos atendimentos para constatação de óbitos, os quais posteriormente foram excluídos. Página 9, linhas 30-35, diz-se que “apenas 9.876 (74,1%) atendimentos apresentaram completude das informações registradas e puderam ser considerados para análise de sobrevida...” mas na descrição de casos foram incluídos todos?

Página 8, linhas 50-53, cita-se que “A maioria dos atendimentos aconteceu prioritariamente no período da manhã”, quando o percentual é de um terço dos casos.

Página 9, linhas 46-58- o texto referente a tabela 2 não esta claro; são apresentados os tempos medianos, sem apresentar a medida de variabilidade (IQR e/ou amplitude de valores).

A notação correta para DP é “DP±XX”

A Tabela 1 apresenta média e mediana. Importante indicar qual a distribuição dos dados e apresentar a medida de tendência central mais adequada, junto a medida de dispersão dos dados.

Na tabela 4, encontra-se significância estatística para “Outros motivos de atendimento”, sendo importante melhor descrevê-los. Poderia ser adicionada em nota de rodapé quais entraram nessa categoria.

O texto “Para melhor compreensão do evento estudado optou-se por realizar o cálculo de associação entre a razão de óbitos por municípios atendidos,” na página 10, linhas 21-25, e “para o cálculo do risco de óbito entre variáveis identificadas como significativas .... foi realizado modelo regressivo univariado (Regressão de Cox)”, deveriam estar na seção métodos, para nos resultados descrever o principal resultado apresentado na tabela.

Discussão

A discussão inicia com a afirmação de que “O presente estudo confirmou o impacto assistencial do SAMU na redução da mortalidade e, consequentemente na sobrevida dos pacientes,” o que de fato não pode ser evidenciado com o estudo proposto e não tem embasamento nos resultados apresentados. Ainda que tenha sido avaliado o tempo transcorrido do chamado ao atendimento, restam duvidas sobre o registro dessa informação, além do que a frase (talvez pelo uso da palavra ´impacto´) dá uma falsa interpretação de que foram comparados pacientes atendidos ou não pela SAMU, sendo a mortalidade menor entre os atendidos pelo serviço de urgência.

Pagina 11 linha 55, a ref 15 aparece entre ( ). O trecho relatado com dados do Datasus não faz relação direta com a pandemia de COVID-19. Dessa forma, nas linhas 51-54, rever a afirmativa de que esses dados “corroboram com o maior número de óbitos e maior tempo de resposta do SAMU em estudo, no ano de 2020 (p<0,001)...” Essa frase, aliás, poderia estar mais destacada nos resultados.

Por fim, importante estabelecer as implicações das limitações para o estudo, especialmente considerando o apontado de falhas nos registros e incompletude e “perda de seguimento”.

Recommendation: Major revision

## Second round comments

Caros autores 

Agradecemos e parabenizamos pelo seu esforço em aprimorar o seu manuscrito, ainda que algumas limitações persistam. Consideramos o artigo aceitável para publicação na RESS, mediante revisões menores. Sugerimos fortemente que seja realizada uma revisão ortográfica, pois encontram-se erros de grafia e concordância.

Segue os itens a serem revistos:

1. No resumo, foi inserido IC95% após o HR, mas sem indicar os valores do limite inferior e superior.

2. Descrição dos métodos foi aprimorada. Cabem ainda algumas observações, descritas a seguir:

2.1 Verificar o trecho “Os dados foram coletados até atingir amostra mínima, entretanto muitos relatórios apresentavam incompletude parcial dos dados, chegando ao número de 13.326 relatórios analisados, dos quais 9.876 estavam com preenchimento completo, representando 5.199 (52,64%) atendimentos do Polo A e 4.677 (47,35%) do Polo B.”Esses dados aparecem antes da definição do tamanho de estudo e cálculo de amostra, sendo mais adequado que o trecho das linhas 10-14 seja inserido após o texto da linha 24 (no mesmo item). Esta é uma limitação importante do estudo que deve ser melhor descrita no parágrafo correspondente na discussão.

2.2 Linha 29 - o valor p representado está incorreto 5% (p-valor > 0,05) e não > 0,005.

2.3 Linhas 37-40: As informações referentes à ética do estudo devem constar unicamente na folha de rosto, conforme modelo disponibilizado.

3. Resultados

3.1 Página 5, linha 5: média de idade ao invés de ´idade média´, pois esse termo remete a um período histórico e não à medida de tendência central dos dados.

3.2 Linhas 22-28: rever a escrita do trecho “Considerando as variáveis com significância estatística para o desfecho óbito na Tabela 1, as quais, motivos de atendimento, ano, recurso móvel e Polo, foi elaborado o Figura 2, que apresenta a análise de sobrevivência, por meio dos testes de Log-rank e comparação global de Breslow (Wilcoxon generalizado), a fim de identificar possíveis diferenças entre as variáveis e os desfechos (óbito/não óbito), uma vez que o desfecho é tempo dependente”. Foi elaborada a Figura 2; O texto “uma vez que o desfecho é tempo dependente” é redundante e desnecessário.

3.3 Necessária revisão de português, pois ainda se encontra erros de grafia e pontuação, como o uso de crases de forma incorreta. Exemplo: “Em relação aos motivos de atendimento, às causas externas apresentaram diferença significativa...”

3.4 Linhas 44-46: “sugerindo que houve um aumento no tempo de resposta em 2020, possivelmente influenciado por fatores como a pandemia da “covid19”. Esse trecho deveria ser deslocado e contextualizado na discussão.

3.5 Linha 47-51, no trecho “a Unidade de Suporte Básico apresentou um tempo mediano de resposta de 9,58 minutos (AIQ: 9), enquanto a Unidade de Suporte Avançado, que lida com atendimentos à (sem crase) vítimas mais graves, teve um tempo mais elevado de 9,88 minutos (AIQ: 9)” foi realizada comparação estatística entre estes valores para fazer essa afirmação? Pois são bem próximos.

3.6 Página 6, linhas 16-17: Inserir o trecho “A Tabela 3 confirmou que o maior número de óbitos ocorreu nos municípios mais distantes, representados pelo Polo B.” no parágrafo anterior (linha 15).

3.7 Linhas 18 - 23: Sugere-se inserir alguns dados numéricos referentes a Tabela 4, pois o texto parece conclusão e não resultado.

3.8 Tabela 1 - Substituir ´Índice de confiança´ por Intervalo de confiança de 95%.

3.9 Figura 2- necessário aprimorar a resolução para melhor visualização. Tabela 3 parece recortada - aprimorar a visualização.

4. Discussão

4.1 Página 6, linhas 38-39: sugere-se remover o termo ´indice` da frase “A análise do tempo por motivos de atendimento demonstrou maior índice de mortalidade...”

4.2 Página 7, linhas 50-51: ajustar a concordância na frase “No âmbito da pesquisa científica, esta pesquisa sobre a sobrevida nos atendimentos realizados pelo SAMU é um dos primeiros com essa magnitude...” seria “...uma das primeiras...”?

Recommendation: Minor revision

## Third round comments

Caros autores

Agradecemos seu esforço em aprimorar o manuscrito e responder aos nossos questionamentos.

Dois itens ainda merecem atenção:

1. Quanto à disponibilidade de dados do artigo, declara-se que “o banco de dados e os códigos de análise utilizados na pesquisa estão disponíveis em https://doi.org/10.1590/SciELOPreprints.10275”, mas não encontrei estes dados no link.

2. A que cálculo se refere a "Razão de óbitos (%)" apresentada na Tabela 3? O valor apresentado não condiz com a razão entre óbito versus não óbitos nas linhas 1,3 e 5, e a constante indicada no título é 1000, e não % (100) como indicado no titulo da coluna. Esse cálculo também não é mencionado no texto dos métodos. Favor rever. 

Recommendation: Minor revision

